# Investigation of creep mechanical characteristics of femoral prostheses by simulated hip replacement

**DOI:** 10.3892/etm.2013.966

**Published:** 2013-02-19

**Authors:** GUANG-YAO LIU, YAN JIN, PENG LI

**Affiliations:** 1 Department of Orthopedics, China-Japan Friendship Hospital, Jilin University, Changchun 130031, P.R. China; 2 Department of Ophthalmology, The Second Hospital of Jilin University, Changchun 130041, P.R. China; 3 Department of Engineering Mechanics, Nanling Campus, Jilin University, Changchun 130022, P.R. China

**Keywords:** hip, artificial prosthesis replacement, stress relaxation, creep, comparative analysis

## Abstract

In order to provide creep mechanical parameters for the clinical application of both traditional and reserved anatomy femoral artificial joint replacements, simulated hip replacement femoral stress relaxation and creep experiments were performed. Twenty-four corpse femoral specimens were obtained, with 8 specimens being randomly assigned to the control group and 8 specimens being randomly assigned to the traditional prosthesis group. Our results showed that the retaining femoral neck prosthesis and traditional prosthesis groups have different stress relaxation and creep mechanical properties.

## Introduction

Due to trauma or disease, the natural joints of >100 million individuals worldwide are replaced each year through the surgical implantation of artificial hip joints (1). Total hip arthroplasty is recognized as one of the most successful and influential orthopedic surgeries of the 20th century, but the service life of an artificial prosthesis is a significant defect. Factors such as prosthesis design, installation and material may affect service life (2). The design and clinical application of an artificial prosthesis has been an important issue for the international research community. Thus far, studies have mainly focused on the clinical application of artificial hip joints and the biomechanics of hip joint replacements (2–16). Nogler *et al* (17) compared the stability of robots and the prosthesis handle of the femoral bone by *in vitro* artificial insertion experiments and showed that insertion by robots cannot improve the initial stability of the prosthesis handle. Halley *et al* (18) implanted a large diameter femoral head when abducens angle of acetabular prosthesis is large and analyzed hip joint stability. The stabilizing effect of the size of the femoral head on the hip joint is mainly dependent on the phase of acetabular prosthesis, while a modular femoral head possessing a sleeve is able to reduce the dislocation of the prosthesis through detailed preoperative design, using a prosthesis with large eccentricity and conservative femoral neck osteotomy. Bevill *et al* (19) adopted a numerical analysis method to investigate the transient hydrodynamic lubrication performance of the system in the crushing movement alone and crushing movement combined with vibration and rotation. The results showed that if an acetabular cup with a relatively large radius or small radial diastema and appropriate geometrical structure was used, the metal hip joint is able to achieve a lubrication state of full film. Dudda *et al* (20) adopted the full numerical analysis method to discriminate the hydrodynamic lubrication problem of the shaft bearing of the hard hip joint and found that the radius of the femoral head and the radial inter-space between the acetabular cup and femoral head had an impact on the thickness of the lubrication film. In the case of steady-state feed motion, Sakagoshi *et al* (21) adopted the finite element and finite difference Newton-Raphson method for the total hip replacement prosthesis system. By solving the Reynolds equation and elasticity equation, the authors found that the bottom of the support layer material of the acetabular cup had less impact on the contact pressure of the shaft bearing surface and elastic deformation.

Previous studies *in vitro* have mainly focused on compression, bending, torsion mechanics and finite element calculations after implantation of the prosthesis, while studies on creep characteristics of the femur after implantation of the prosthesis *in vitro* have been rarely reported. Using the finite element model, Bevill *et al* (19) simulated the situation of prosthesis wearing under a million times the gait load, and analyzed the factors which affected wearing, such as polyethylene coating thickness and femoral head prosthesis size, in order to improve the design of the prosthesis. The results demonstrated that the creep behavior of polyethylene accounted for 10–50% of the total wearing of acetabular liners, which mainly occurred in the early stages. With the same model, the results of Dudda *et al* (20) showed that the movements of the hip joint were the main guiding forces of prosthesis pressure changes, stress on the joints and partial dislocation, and in some cases even caused dislocation. The authors found that the risk of dislocation was highest when changing from a low sitting position to a standing position, six times higher than that from a bending over position to a standing position. Sakagoshi *et al* (21) used finite element analysis to show that after hip resurfacing arthroplasty, walking in normal and osteoporotic conditions would not generate the stress that could cause fractures in the proximal femur, therefore demonstrating that hip resurfacing combined with concurrent femoral neck fracture may be the result of multiple complex factors. Previous studies mainly used *in vitro* experiments to calculate compression, bending, twisting and finite elements of prostheses, while the femoral creep characteristics of *in vitro* prostheses have rarely been reported.

During the process of total hip arthroplasty, the femoral neck needs be removed, and thus the force on the upper section of the femur is affected. The femur is a biological viscoelastic material, and its stress relaxation creep characteristics meet the need of the individual’s physiological functions to maintain body balance and stability. Prosthetic replacement may cause the loss and damage of the cancellous bone, bone marrow and cartilage at the implantation location and impact on the stress relaxation and creep properties of the femur. Considering the needs for clinical practice, this study was designed to simulate total hip arthroplasty, and stress relaxation and creep experiments of the femur were performed before and after the retained femoral neck prosthesis (made in Germany) and the traditional prosthesis (Beijing Prussia Co., Beijing, China) were inserted. Data and curves of stress relaxation, creep characteristics, stress, variation rule of strain and time of the specimens on three groups were also obtained to compare and analyze the fixed-effects of the two artificial joints based on the creep characteristics of stress relaxation. The stress relaxation creep characteristics of the femur before and after hip replacement were obtained experimentally to provide visco-elastic parameters for artificial hip joint arthroplasty research.

## Materials and methods

### Samples

In this experiment, 24 corpse femur specimens of healthy individuals aged 22–30 years old in China were provided by the Department of Anatomy, Norman Bethune Medical University. This study was conducted in accordance with the declaration of Helsinki. This study was conducted with approval from the Ethics Committee of The Second Hospital of Jilin University. Written informed consent was obtained from all families. Femur anatomical specimens on the left and right were obtained within 2 h of death and wrapped with a saline-soaked gauze. The specimens were put into a tightly closed plastic bag and stored in a −20°C refrigerator.

### Grouping

The specimens were removed from the refrigerator before the experiment and thawed at room temperature. Twenty-four samples of left and right femurs were taken. Four specimens were randomly selected from the left and right side and considered as the control group. A further eight specimens on the left femoral neck were randomly selected and treated with the artificial joints for the titanium retained femoral neck prosthesis. Another eight samples were randomly selected for the right femoral neck and treated with the traditional cobalt-chromium-molybdenum prosthesis.

### Traditional prosthesis

The femur was mounted on a fixed platform at the top of the lesser trochanter to 1.5 cm at the bottom of the femoral neck osteotomy lungi outside of the greater trochanter. The femoral head and most of the femoral neck were removed and the cancellous bone of the distal femur was excavated to determine the location of the medullary cavity, remote from the greater trochanter through the lateral canal, and the internal debris was removed with a reamed intramedullary nail file. A suitable prosthesis was selected for biological fixation and the neck was maintained forward by 10–15°.

### Anatomical prosthesis retained femoral neck

The femur was fixed on a platform and vertical osteotomy was performed on the femoral head following the femoral neck. The lateral line from the greater trochanter osteotomy base is usually 1.5 cm, and the femur head was removed. A pore was opened on the central side of the lesser trochanter, and an appropriate camera pulpi file was selected to expand the medullary cavity, and the prosthesis was placed.

### Load relaxation

The length and diameter of the femur were measured by a steel ruler and reading microscope. The length and diameter of the traditional cobalt-chromium-molybdenum prosthesis were 372.6–428.6 and 26.86–27.42 mm, respectively, while the length of and diameter of artificial joints for the titanium retained femoral neck prosthesis were 378.2–438.6 and 26.82–28.83 mm, respectively.

**Figure 1:**
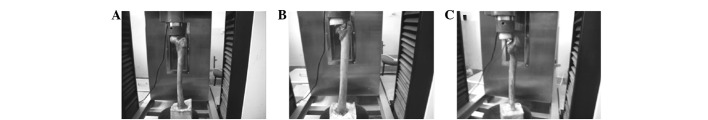
Load relaxation experiment images of the specimens in three groups. (A) Load relaxation experiment image of the control. (B) Load relaxation experiment image of the traditional type of prosthesis implantation group. (C) Load relaxation experiment image of the preservation of femoral neck prosthesis implantation group.

The original size of the sample was input to the computer controlling the machine. The specimen was placed on the working table of the test machine, the top of samples and department head of the testing machine were in contact. In this study, each sample was pre-treated and adjusted for experiments as described previously (18). In order to maintain sample moisture, the samples were wrapped in a saline-soaked gauze and periodically sprayed with saline with a pH value of 7.4. The testing machine was equipped with a 35–250°C ambient temperature box, which automatically adjusted the temperature and kept the temperature constant. This experiment was performed at 36.5±0.5°C at a specimen rate of 30 mm/min. When the displacement of the control group reached 1.11 mm, displacement of the anatomic prosthesis group reached 1.12 mm, and that of the traditional prosthesis fixation reached 1.13 mm. When the load of each group reached 1 kN and the displacement constant was kept the same, the load constantly dropped with the change of time. From the set computer program, time t (0) began to collect data, a datum was collected every 0.6 sec, collected 10 times, followed by a datum acquisition every 10 sec, collected 40 times, a datum collected every 136 sec, collected 50 times, and 100 data were collected over 7200 sec. The experimental data and curves were printed out automatically. The load relaxation experiment controls are shown in Fig. 1A, the load relaxation of the traditional type of prosthesis implantation group is shown in Fig. 1B and the load relaxation of the preservation of femoral neck prosthesis implantation group is shown in Fig. 1C.

### Creep test

The size measurement of sample, sample size, sample pre-transfer treatment, test temperature, sample moisture method and sample installation were the same as those of the stress relaxation experiments. The sample was applied to the specimen loading at a 10 N/sec rate. When the displacement of the normal control group reached 1.12 mm, the displacement of the traditional prosthetic group reached 1.12 mm and that of the traditional prosthesis fixation reached 1.12 mm. When the load of the normal control group reached 0.99 kN, the load of the anatomic prosthesis group reached 0.98 kN and that of the traditional prosthesis group reached 1.0 kN and the load was kept constant. The displacement was increased with the change of time. From the set computer program, time t (0) began collecting data, a datum was collected every 0.6 sec, collected 10 times, followed by data acquisition every 10 sec, a datum was collected every 136 sec, collected 50 times, 100 data were collected over 7200 sec. The experimental data and curves were printed out automatically.

### Statistical analysis

The SPSS 16.0 (Chicago, IL, USA) package was used for data analysis, the results are expressed in mean ± SD, single-factor analysis of variance was used to analyze the experimental data, Scheffe’s method was used to compare the difference between groups. P<0.05 was considered to indicate a statistically significant result.

## Results

### Relaxation curves

**Figure 2:**
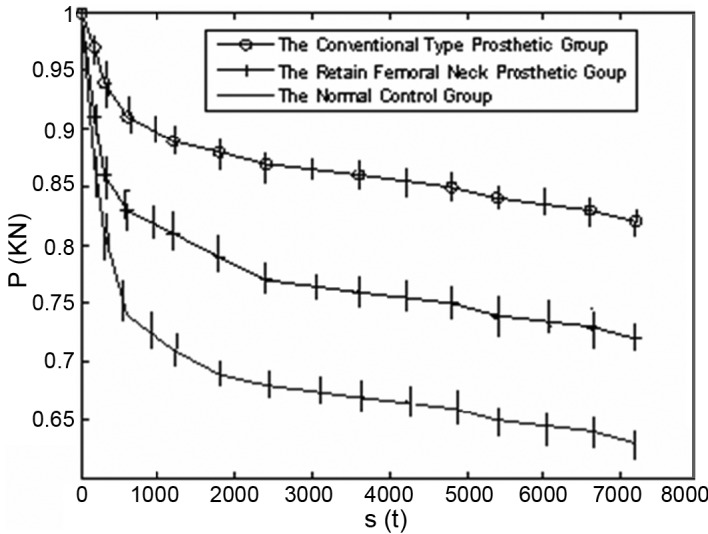
Load relaxation curves of the specimen in each group.

The relaxation curves for the loading of specimens of the two groups are shown in Fig. 2. The load relaxation curve showed logarithmic change, and when the time reached 7200 sec, the load relaxation curve tended to level out. When the time reached 7200 sec, the load of the control group was decreased by 0.25±0.018 kN, the load of the traditional prosthesis group was decreased by 0.18±0.020 kN, while that of the retained prosthesis group was decreased by 0.22±0.016 kN. By comparison with t-test of paired data, the load decrease of the control group at 7200 sec was significantly increased compared with that of the other two groups (P<0.05), while the reduction of the load of the traditional prosthesis group was significantly less than that of the anatomic pros-thesis group (P<0.05).

### Creep curves

**Figure 3:**
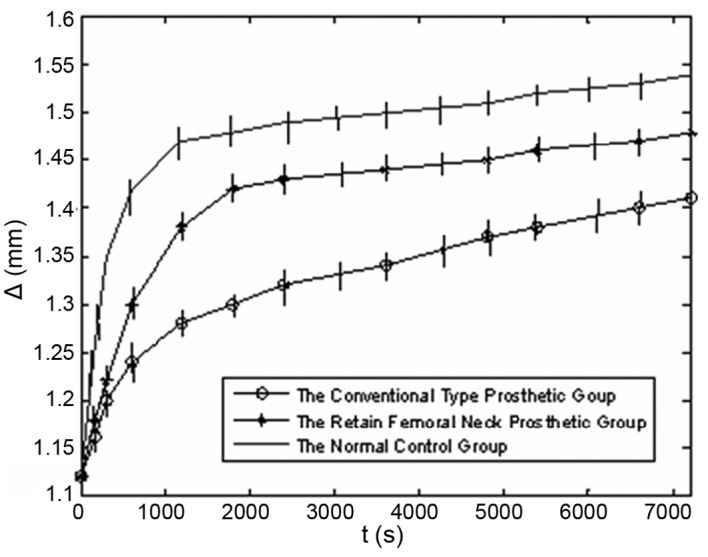
Creep curves of the specimen in each group.

The creep curves of the 8 specimens of the two groups are shown in Fig. 3. The creep curve showed exponential change, and when the time reached 7200 sec the load relaxation curve tended to level out. The 7200 sec increased displacement of the traditional prosthesis group was 0.29±0.016 mm, the increased displacement of the retained prosthesis group was 0.36±0.18 mm and that of the normal control group was 0.42±0.14 mm. By comparison with t-test of paired data, the increased displacement of the control group at 7200 sec was significantly increased compared with that of the other two groups (P<0.05), while the increased displacement of the traditional prosthesis group was significantly less than that of the anatomic prosthesis group (P<0.05).

## Discussion

The femur is a viscoelastic solid material, and the characteristics of such biological materials include stress relaxation and creep. Stress relaxation and creep experiments are important methods to study the simulated stability of total hip arthroplasty prostheses. This experiment compared the stress relaxation and creep rheology of simulated total hip arthroplasty, the traditional prosthesis implantation group and the anatomic femoral prosthesis group. The stress relaxation, creep characteristics, stress, variation rule of strain and time of the specimens of the three groups were obtained. The details of the experimental methods used are as follows: i) The stress relaxation and creep characteristics of the two specimens of the traditional prosthesis group and anatomic prosthesis group at the same experimental set speed, the same constant strain, the same constant stress and the same test temperature were recorded. The test time conditions were 7200 sec. ii) The experimental design was a comparative analysis for randomized specimens. iii) Each specimen was independent, and the experimental data error was evaluated by statistical analysis and t-test methods. The stress relaxation, creep characteristics, stress, variation rule of strain and time of the specimens of the three groups were obtained and the viscoelastic characteristics of the specimens of the two groups were quantitatively compared. This experiment was based on mathematical statistical methods, and quantitatively compared the stress relaxation and creep properties of the two groups to assess the rheological indicators of the specimen on the traditional implantation group and anatomic prosthesis group. Due to body age, health status, occupation and individual differences, the experimental data varied to a certain degree, but also revealed the stress relaxation and creep properties of the specimens of the traditional implantation group and anatomic prosthesis group.

Our experimental results showed that the load decrease of the control group at 7200 sec was significantly increased compared with that of the other two groups (P<0.05), while the reduction of the load of the traditional prosthesis group was significantly less than that of the anatomic prosthesis group (P<0.05). The increased displacement of the control group at 7200 sec was significantly increased compared with that of the other two groups (P<0.05), while the increased displacement of the traditional prosthesis group was significantly less than that of the anatomic prosthesis group (P<0.05). The load relaxation curve showed logarithmic change, while the creep curve showed exponential change.

Load relaxation and creep rapidly changed in the first 600 sec, and after the load slowed down, displacement slowly increased. When 7200 sec was reached, the load relaxation and creep curves tended to level out. Stress relaxation and creep rapidly changed in the first 600 sec in the experiment due to the inherent femur tissue swelling caused by water pressure and partial pressure spilling out more rapidly, with the femoral organization water outflow, its inherent pressure and expansion pressure were reduced, and therefore the latter part of strain was slowly changed.

The reduced load of traditional prosthesis is too large at 7200 sec, and is not conducive to resist the constant strain acting on the specimen, which indicates that after implantation of the prosthesis, under the effect of constant displacement, stress shielding of the samples was significant. It is not conducive to stability after the implantation of the prosthesis. The displacement of the retained femoral neck group was large at 7200 sec, which indicated that the capacity of its resistance to a constant compression load was large. The femur tissue was longitudinally elongated under constant load, and may play a limited role in the sinking of the prosthesis and aid the stability of the femur after the implantation of the prosthesis. The amount of displacement of the traditional prosthesis group was less, and it is not conducive to the stability of the femur after implantation of the prosthesis.

The load relaxation of bone and creep characteristics are correlated with moisture, collagen and elastic fiber content contained in bone tissue and the direction of arrangement. In this study, in the conventional prosthesis group, the femoral head and femoral neck were removed, bone marrow in the vertical interface of the femur was severely damaged and moisture of bone tissue was lost. Collagen and elastic fibers were damaged, so the amount of its increased displacement at 7200 sec was reduced, and its inherent rheological properties were changed. Due to retaining the femoral neck of the anatomical femoral prosthesis group, damage to the intramedullary cavity was less than that of the traditional prosthesis group, so the increased displacement at 7200 sec was greater than that of the traditional prosthesis implantation group. This femoral prosthesis design met biomechanical requirements.

After the total hip replacement, the joint stress was spread to the femur through the prosthesis, and the transfer of the stress changed, resulting in stress shielding and stress concentration. Beulah *et al* (22) used the finite element model to study the new prosthesis design, hexagonal cross-section with low elastic modulus, and concluded that this form would be more conducive to reducing the stress shielding and enhancing the fixation of the prosthesis. Certain researchers reconstructed the finite element analysis model with metal-metal hip resurfacing arthroplasty, virtually loaded and simulatively calculated the model to compare and analyze the femoral stress distribution after surgery. The results showed that after surgery, stress was mainly concentrated in the medial side of the femoral neck and the junction of the head and neck, and stress shielding in the lower sclerotin of femoral prosthesis was observed. After the hip resurfacing arthroplasty, stress concentration and stress shielding had some contact with femoral and neck fracture (23). Fouad (24) used the finite element method to demonstrate that small changes of the material properties of artificial femoral head may have a great impact on the stress distribution of the hip joint.

Arabmotlagh *et al* (16), based on the theory that a low elastic modulus femoral prosthesis is less intrusive to normal biomechanics, presented a prosthesis design: the no supportable neck, in which titanium was used to prevent fatigue fracture, while carbon fiber, whose elastic modulus is close to that of bone, was applied to the leg in order to optimize mechanical transmission. Biomechanical studies have shown that the rigidity of the handle and the handle diameter is proportional to the fourth power, while the hollow prosthesis significantly reduces stiffness. The stress shielding effect of the carbon fiber prosthesis is smaller, but even so there remains a strong stress shielding on the lateral proximal femoral bone, which indicates that the premises of stress shielding is an extremely difficult problem to solve, as its axial and torsional loads on the femur cross-sectional strain from the area is greater than titanium alloy and stainless steel prosthesis. This showed that the proximal load was increased, and suggested that the proximal prosthesis design of low modulus of elasticity is particularly important, otherwise the risk of proximal loosening will greatly increase. Metal with high strength and high modulus of elasticity may cause serious stress shielding, so a material with low elastic modulus and high toughness will become the new selection for femoral stem prosthesis. Carbon fiber composite materials, titanium alloy and ceramic materials are also likely to be advantageous in this area. Polyethylene as a prosthetic friction surface material has wear debris and raises a series of questions. The wear resistance of a metal-metal joint surface has evident advantages, and as long as the manufacturing process achieves a certain level, it is a promising option to replace a polyethylene-metal joint surface (18).

Both the design and material biomechanical effects of the prosthesis are critical. The rigidity of a prosthesis is correlated with the elastic modulus, cross-sectional area and shape of the material. However, the stress-shielding effect of a large and rigid prosthesis handle is not necessarily strong as the stress-shielding is the result of many factors. The correlation is mainly attributed to the match of the proximal prosthesis. In addition, the elastic modulus relatively close to the cortical bone may elastically reduce the role of stress shielding (14).

Previous studies (2–5) mainly simulated the compression and bending experiments of the artificial joint implanted into the femur, while the present experiment was designed to stimulate total hiparthroplasty, and was implanted with the prosthesis retained femoral neck made in Germany and traditional prosthesis made by Beijing Prussia.

Personalized prosthesis research is imperative, and since prostheses used in clinical practice do not consider the geometry of the prosthesis into the femur with the matching geometry, it is difficult for the same type of prosthesis to meet the needs of all patients, and the prosthesis results in subsidence or loosening. Individual prosthesis design should change with the patient and meet the need of the patient. Individual prosthesis has broad development prospects.
